# Monoacylglycerol Lipase Regulates Fever Response

**DOI:** 10.1371/journal.pone.0134437

**Published:** 2015-08-19

**Authors:** Manuel Sanchez-Alavez, William Nguyen, Simone Mori, Gianluca Moroncini, Andreu Viader, Daniel K. Nomura, Benjamin F. Cravatt, Bruno Conti

**Affiliations:** 1 Department of Chemical Physiology, The Scripps Research Institute, 10550 N. Torrey Pines Rd., La Jolla, CA, 92037, United States of America; 2 Dipartimento di Scienze Cliniche e Molecolari, Università Politecnica delle Marche, Ancona, Italy; 3 Department of Nutritional Sciences and Toxicology, University of California, Berkeley, CA, 94720, United States of America; University of Insubria, ITALY

## Abstract

Cyclooxygenase inhibitors such as ibuprofen have been used for decades to control fever through reducing the levels of the pyrogenic lipid transmitter prostaglandin E2 (PGE_2_). Historically, phospholipases have been considered to be the primary generator of the arachidonic acid (AA) precursor pool for generating PGE_2_ and other eicosanoids. However, recent studies have demonstrated that monoacyglycerol lipase (MAGL), through hydrolysis of the endocannabinoid 2-arachidonoylglycerol, provides a major source of AA for PGE_2_ synthesis in the mammalian brain under basal and neuroinflammatory states. We show here that either genetic or pharmacological ablation of MAGL leads to significantly reduced fever responses in both centrally or peripherally-administered lipopolysaccharide or interleukin-1β-induced fever models in mice. We also show that a cannabinoid CB1 receptor antagonist does not attenuate these anti-pyrogenic effects of MAGL inhibitors. Thus, much like traditional nonsteroidal anti-inflammatory drugs, MAGL inhibitors can control fever, but appear to do so through restricted control over prostaglandin production in the nervous system.

## Introduction

Fever is a physiological response to pathological conditions such as infection, malignancy, or severe tissue damage. Fever typically occurs when cells of the immune system respond to exogenous or endogenous insults by producing and releasing specific cytokines that ultimately lead to the production of the pyrogenic prostaglandin E2 (PGE_2_) in either the brain vasculature or peripheral tissues [1,2]. PGE_2_ elicits febrile responses largely through stimulating prostaglandin E receptor 3 (EP3) on neurons of the medial and the median preoptic nuclei (MPO and MnO, respectively) of the preoptic area (POA), leading to disinhibition of thermogenic neurons in caudal brain regions and activation of thermoregulatory effectors to increase heat production and reduce heat loss [3–16]. Indeed, PGE_2_-lowering cyclooxygenase (COX) inhibitors, such as aspirin and ibuprofen, have been used for over a century as fever-lowering agents.

PGE_2_ is synthesized from arachidonic acid (AA) precursor pools, which have generally been thought to derive from membrane phospholipids by the action of phospholipase A2 (PLA_2_) enzymes [17,18], although alternative pathways have been considered in select biological systems [19,20]. We recently showed that brain prostaglandins principally originate from an AA source provided by monoacylglycerol lipase (MAGL)-mediated hydrolysis of the endocannabinoid 2-arachidonoylglycerol [21]. Mice null for MAGL (*Mgll* ^*-/-*^) or mice treated with the MAGL inhibitor JZL184 show elevations in brain 2-AG, and reductions in brain AA and prostaglandins under basal conditions and in multiple inflammatory and neurodegenerative mouse models, leading to cannabinoid receptor-independent attenuation of neuroinflammation and neuroprotection [21–23]. Mice deficient in the 2-AG biosynthetic enzyme diacylglycerol lipase-alpha (DAGLα) also exhibit reductions in brain AA [24], and inhibitors of DAGLβlower AA and PGE_2_ in peritoneal macrophages in a manner that is complementary to the ablation of cytosolic phospholipase A2 (cPLA2 or PLA2G4A) [25].

Recent studies have suggested that MAGL inhibitors may be used to treat various pathologies, through either enhancing endocannabinoids, lowering eicosanoids, or both, to alleviate pain, inflammation, anxiety, and depression [26,27]. Here, we have investigated the potential of MAGL blockade to attenuate both centrally and peripherally-induced fever responses in mice.

## Methods

### Mice

All procedures were approved by Institutional Animal Care and Use Committee of the Scripps Research Institute and were done in accordance to NIH Guide for the Care and Use of Laboratory Animals. *Mgll* ^*-/-*^ and *Mgll* ^*+/+*^ mice were previously described by us and were originally obtained from Texas A&M Institute of Genomic Medicine and from Joseph Bonventre's laboratory at Brigham and Women's Hospital. Null mice and wild type littermates were obtained by crossing *Mgll* ^*-/+*^ heterozygous animals. All experiments were carried out on adult 3–5 month old male mice maintained at constant environmental conditions of 25 ± 0.5°C and 37 ± 2% humidity with water and food provided ad libitum unless specified, and subjected to a 12:12 hrs light:dark cycle with lights on at 7 AM.

### Telemetry

Telemetry was performed as previously described by us [28–31]. Briefly, mice were anesthetized with isoflurane (induction 3–5%, maintenance 1–1.5%) and surgically implanted with radiotelemetry devices (TA-F10, Data Sciences, St. Paul, MN) into the peritoneal cavity for core body temperature (CBT) and activity. Following surgical implantation and appropriate wound closure, the animals were allowed to recover for 2 weeks and then submitted to freely moving telemetry recordings. Mice were individually housed in a plexiglas cage in a room maintained at 25 ± 0.5°C. The cages were positioned onto the receiver plates (RPC-1; Data Sciences, St. Paul, MN) and radio signal from the implanted transmitter were recorded every 5 minutes with fully automated data acquisition system (Dataquest ART, Data Sciences, St. Paul, MN).

### Chemicals and Injections

Bacterial lipopolysaccharides (LPS) (0127:B8, Sigma, St. Louis, MO) were administered i.p. using a volume of 100–200 μl per mouse at a dose of 100 μg/kg (~3 μg/mouse), a dose previously demonstrated by us and others to induce fever [28,32].

Recombinant IL-1β (R&D Systems) was administered centrally in the preoptic area (POA through a cannula previously implanted at the following stereotactic coordinates: (anterior-posterior [AP] from bregma = 0.38 mm, lateral [Lat] = midline, ventral [V] = 3.8 mm, cannula 26 GA, 10 mm length). Following a 7 day recovery period, single caged animals received 0.5 μl of vehicle (aCSF, artificial cerebrospinal fluid) or of 500 pg of recombinant IL-1β (R&D Systems Inc, Minneapolis, MN) in aCSF using an injector through the cannula connected to plastic tubing and a microsyringe using an injector (33 GA, protruding 0.4 mm beyond the tip of the cannula, total length 10.4 mm) as previously described by us [28,30].

JZL184 (Cayman Chemicals, Ann Arbor, MI) was dissolved in ethanol, followed by addition of Emulphor-620 (Sanofi-Aventis, Bridgewater, NJ), and diluted with 0.9% saline to form a vehicle mixture of ethanol-Emulphor-saline in a ratio of 1:1:18 and was administered i.p. at 40 mg/kg, a dose previously shown to exert full inhibition of MAGL [21]. We demonstrated that the ethanol-Emulphor-aCSF (1:1:18) solution alone does not induce nor prevent fever (not shown).

Rimonabant (SR141716) (Cayman Chemical Co, Ann Arbor, MI) was injected i.p. at a dose of 1 mg/kg 30 min before inhibitors as previously described[33].

### Statistics

Values are mean ± standard error of the mean (SEM). Each data point, in each condition, represents the mean of data collected from at least 6 mice. Longitudinal data on temperature acquired and compared using Repeated Measures ANOVA, followed by Newman-Keuls post-test (P<0.05). Multiple regression analysis was performed for all the longitudinal data. Post-hoc analyses (Tukey LSD **p**<0.05) between vehicle and inflammagen treated groups was determined. For these analyses, **P**-value was set at **p**<0.05 to determine the levels of statistical significance.

## Results and Discussion

### MAGL-deficient mice show no differences in normal core body temperature profile

**Fig 1:**
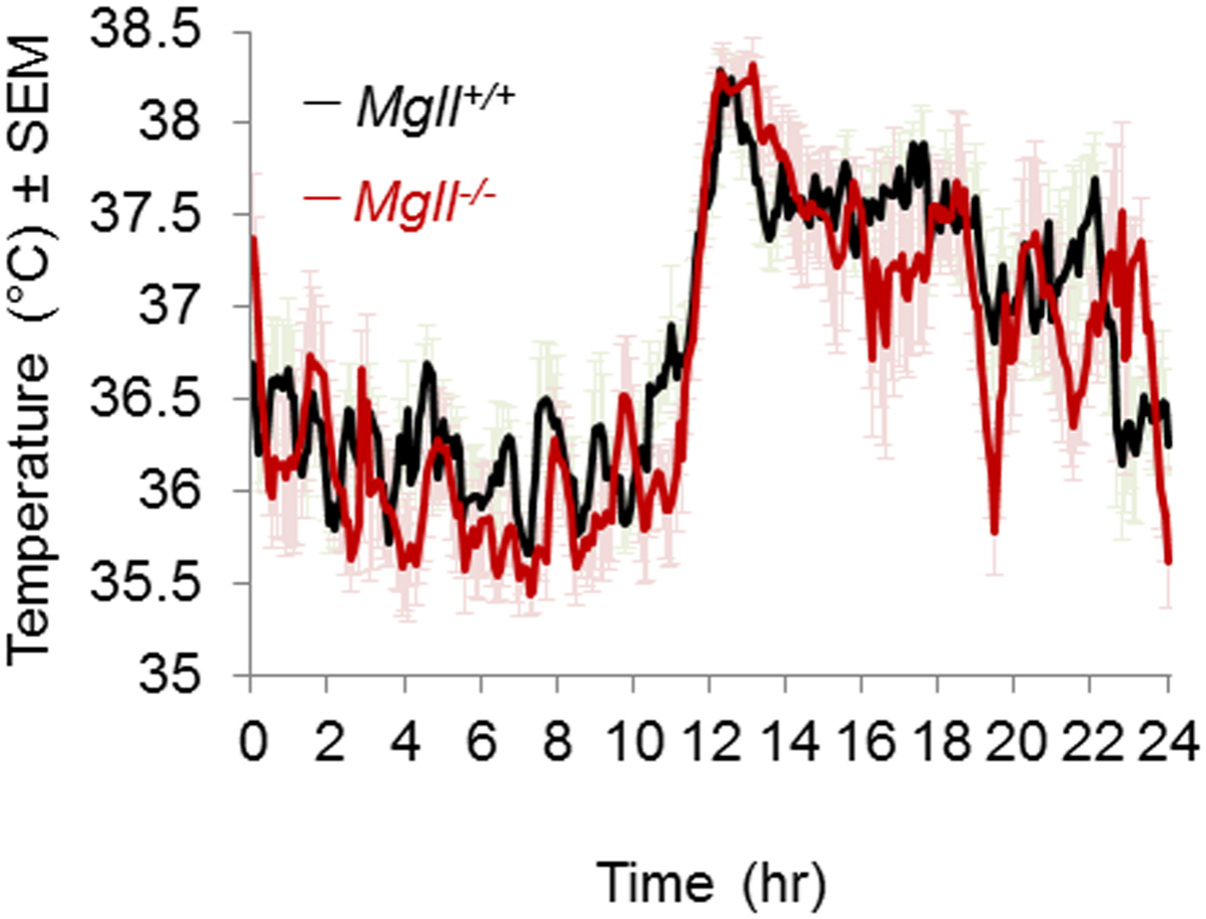
*Mgll* ^*-/-*^ and *Mgll* ^*+/+*^ mice have similar core body temperature profiles. CBT profile of *Mgll* ^*-/-*^ and *Mgll* ^*+/+*^ male mice over 24 hrs. No statistically significant differences were observed across genotypes. Data are shown as mean ± sem, n = 6 mice per group, p>0.05.

Before investigating the possible role of MAGL in fever, we compared the profile of core body temperature (CBT) of *Mgll* ^*-/-*^ and *Mgll* ^*+/+*^ mice. No difference in CBT was observed across genotypes over a 24 hour period of recording (Fig 1). Both groups of animals showed similar and normal CBT profiles in the dark (active part of the day, 12 to 24 hrs), in the light (resting part of the day, 0 to 12 hrs) and during the transitions between phases. This indicates that MAGL is not required for the maintenance of the basal CBT and temperature homeostasis and identify *Mgll* ^*-/-*^ mice as a suitable model to investigate the role of MAGL in fever.

### Genetic and pharmacological ablation of MAGL attenuates peripherally induced fever response

**Fig 2:**
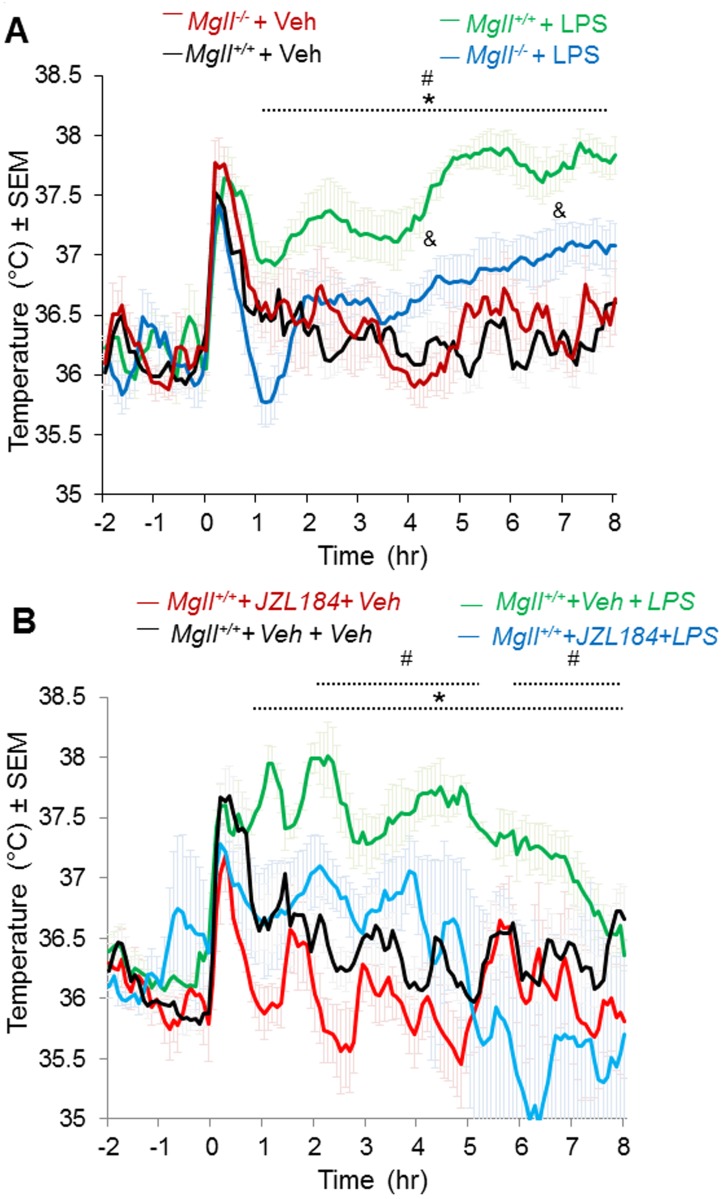
Genetic or pharmacological ablation of MAGL reduces fever response in peripheral LPS-induced fever model. **(A)** CBT profile following i.p. injection of LPS (100 μg/kg) or vehicle (Saline) of *Mgll* ^*+/+*^ mice treated i.p. with JZL184 (40 mg/kg). *p<0.05, *Mgll* ^*+/+*^+ Veh vs. *Mgll* ^*+/+*^ + LPS; ^#^p<0.05, *Mgll* ^*+/+*^+ LPS vs. *Mgll* ^*-/-*^ + LPS; ^&^p<0.05, *Mgll* ^*+/+*^ + Veh vs. *Mgll* ^*-/-*^ + LPS. **(B)** CBT profile of *Mgll* ^*-/-*^ and *Mgll* ^*+/+*^ mice following i.p. injection of LPS (100 μg/kg) or vehicle (Saline) as indicated. Injection was performed at time 0. Data are shown as mean ± sem, n = 6 mice per group, *p<0.05, *Mgll* ^*+/+*^+ Veh + Veh vs. *Mgll* ^*+/+*^ + Veh + LPS; # p<0.05, *Mgll* ^*+/+*^+ Veh+ LPS vs. *Mgll* ^*+/+*^+ JZL184 + LPS.

We next tested whether a peripherally induced fever response could be mitigated upon ablation of MAGL. We induced a fever response in mice by i.p. injection of the exogenous pyrogen lipopolysaccharide (LPS) (100 μg/kg), leading to prolonged and elevated CBT. *Mgll* ^*-/-*^ mice showed significantly attenuated fever responses compared to LPS-treated *Mgll* ^*+/+*^ mice (Fig 2A). We next used the MAGL inhibitor JZL184 [34] to test whether pharmacological blockade of MAGL also affected fever responses. We treated mice with a dose JZL184 (40 mg/kg. i.p., 1 hr before LPS injection) that we have previously shown to produce complete inhibition of MAGL *in vivo*, leading to profound elevations in brain 2-AG and suppression of brain AA and prostaglandins [21]. We found that JZL184 significantly reduced CBT and fever response elicited by LPS, compared to vehicle-treated LPS-administered controls (Fig 2B).

### Genetic and pharmacological ablation of MAGL attenuated centrally-mediated fever response

**Fig 3:**
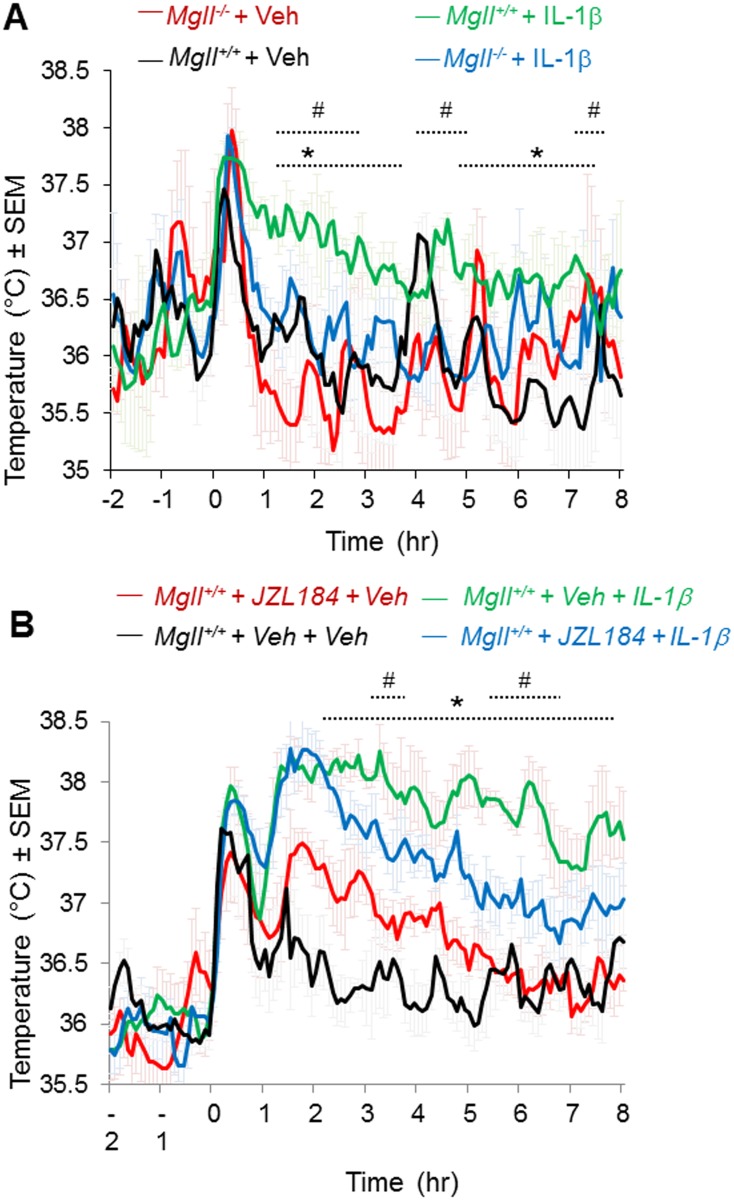
Genetic or pharmacological ablation of MAGL reduces fever response in central IL-1β-induced fever model. **(A)** CBT profile following icv injection into POA of IL-1β (500 pg/0.5 μl) or aCSF of *Mgll* ^*+/+*^ mice treated i.p. with JZL184 (40 mg/kg). *p<0.05, *Mgll* ^*+/+*^ + Veh vs. *Mgll* ^*+/+*^ + IL-1β; ^#^p<0.05 *Mgll* ^*+/*^ + IL-1β vs. *Mgll* ^*-/-*^+ IL-1β. **(B)** CBT profile of *Mgll* ^*-/-*^ and *Mgll* ^*+/+*^ mice following icv injection into POA of IL-1β (500 pg/0.5 μl) or aCSF as indicated. Injection was performed at time 0. Data are shown as mean ± sem, n = 6 mice per group. *p<0.05, *Mgll* ^*+/+*^+ Veh + Veh vs. *Mgll* ^*+/+*^+ Veh + IL-1β; ^#^p<0.05 *Mgll* ^*+/+*^+ Veh+ IL-1β vs. *Mgll* ^*+/+*^+ JZL184 + IL-1β.

To examine whether the observed fever-reducing effects were due to modulation of central pyrogens, we next examined whether genetic or pharmacological ablation of MAGL was capable of attenuating centrally-induced fever in mice through POA administration of the endogenous pyrogen interleukin-1β (IL-1β) (500 pg/0.5 μl). We show that either *Mgll* ^*–/–*^(Fig 3A) mice or mice treated with JZL184 (40 mg/kg, i.p., 1 hr before IL-1βadministration) (Fig 3B) display significantly reduced IL-1β-mediated CBT compared to vehicle-treated or *Mgll* ^*+/+*^ control mice.

### The anti-pyrogenic effects of MAGL inhibitors are independent of CB1 cannabinoid receptor activity

**Fig 4:**
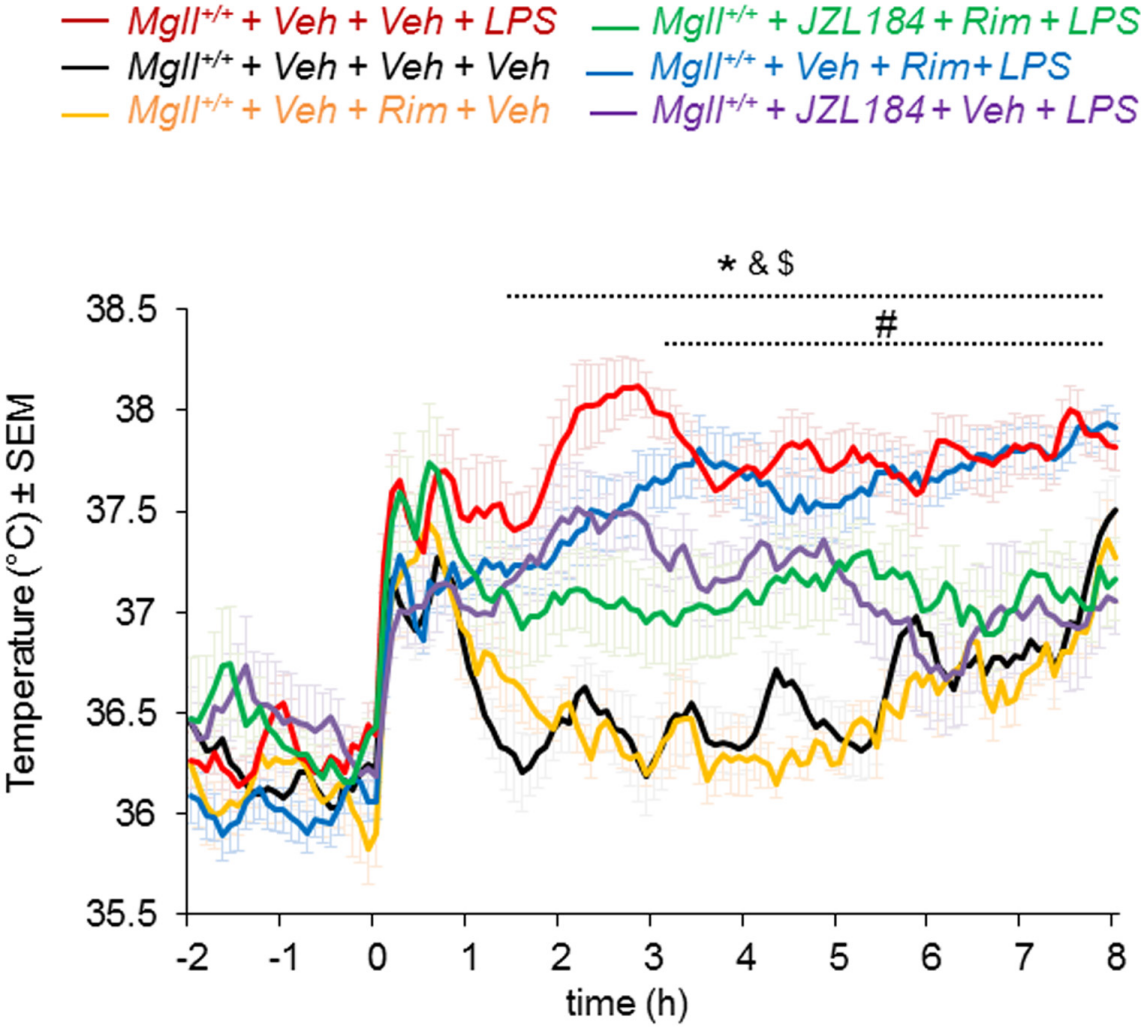
Anti-pyrogenic effects of MAGL inhibitors are independent of CB1 cannabinoid receptor activity. CBT profile following i.p. injection of LPS (100 μg/kg) of *Mgll* ^*+/+*^ mice receiving i.p. injection of rimonabant (1 mg/kg) and/ or JZL184 (40 mg/kg). Rimonabant did not affect the hypothermic effects of JZL184. Data are shown as mean ± sem, n = 6 mice per group, *p<0.05, *Mgll* ^*+/+*^+ Veh + Veh + Veh vs. *Mgll* ^*+/+*^+ Veh + Veh + LPS; ^#^p<0.05, *Mgll* ^*+/+*^+ Veh + Veh + LPS vs. *Mgll* ^*+/+*^+ JZL184 + Veh + LPS; ^&^p<0.05, *Mgll* ^*+/+*^+ Veh + Rim + LPS vs. *Mgll* ^*+/+*^+ Veh + Rim + Veh; ^$^p<0.05, *Mgll* ^*+/+*^+ Veh + Rim + LPS vs. *Mgll* ^*+/+*^+ JZL184 + Rim + LPS. p>0.05 (NS) *Mgll* ^*+/+*^+ Veh + Veh + Veh vs *Mgll* ^*+/+*^+ Veh + Rim + Veh, *Mgll* ^*+/+*^+ JZL184 + Rim + LPS vs *Mgll* ^*+/*^ + JZL184 + Veh + LPS, *Mgll* ^*+/+*^+ Veh + Veh + LPS vs *Mgll* ^*+/+*^+ Veh + Rim + LPS.

Since endocannabinoids have been shown to participate to hypothermic responses via activation of CB1 receptors [35], we next tested whether the anti-pyrogenic effects of MAGL inhibitors were dependent on the central CB1 cannabinoid receptor. Pre-treatment of mice with a cannabinoid receptor type 1 (CB1) antagonist rimonabant (RIM) (1 mg/kg, i.p.) did not alter the anti-pyrogenic effects of JZL184 in LPS-treated mice (Fig 4). These data indicate that the anti-pyrogenic effects of MAGL inhibitors are likely due to reductions in brain PGE_2_.

## Conclusion

Fever is an increase of core body temperature that is regulated centrally and occurs when PGE_2_ binds to the EP3 receptor on hypothalamic neurons that control temperature homeostasis. PGE_2_, like other eicosanoids, is synthesized by cyclooxygenase (COX)-mediated metabolism of arachidonic acid (AA). Indeed, COX inhibitors are widely utilized and effective anti-pyretic drugs (e.g. [36]). Although phospholipases such as cPLA2 have been thought to be the dominant driver of AA for prostaglandin production, cPLA2-deficient mice showed unaltered prostaglandin content in the brain under basal conditions [37], pointing to the existence of alternative pathways that produce AA for eicosanoid synthesis in the nervous system. Recent studies have identified MAGL as a primary regulator of AA and prostaglandin production in mouse brain, and MAGL blockade leads to reduced pro-inflammatory eicosanoids in various neuroinflammatory and neurodegenerative disease models [21–23]. Here, we extend these findings to show that pharmacological or genetic ablation of MAGL reduces fever responses in both peripherally and centrally mediated mouse fever models.

While we show here that MAGL inhibition leads to substantial suppression of the fever response in both the peripheral LPS and central IL-1β fever models, we note that MAGL inhibitors do not completely suppress fever in these models, which contrasts with the full suppression observed with COX inhibitors [38,39], suggesting the existence of other pools of AA that may contribute to PGE_2_ production. Indeed, we previously showed that cPLA2-deficient mice also exhibit a modest reduction in brain prostaglandin levels under LPS challenge and that MAGL inhibition in LPS-stimulated cPLA2-deficient mice additively reduced brain PGE_2_ levels beyond MAGL or cPLA2 ablation alone [21]. Thus, it will be of future interest to determine whether MAGL and cPLA2 dual blockade fully suppresses fever responses in the models described here. Another unanswered question is whether there may be enzymatic diversification in enzymes such as MAGL, cPLA2, or other activities that drive AA release for PGE_2_ synthesis in a cell type or brain region-specific manner. The generation of neuron-specific or microglial-specific *Mgll* ^*-/-*^ mice may be able to address these questions. It would also be important to examine fever responses following dual blockade of MAGL and FAAH, which has been shown, for certain behavioral processes, to produce greater effects than disruption of either single enzyme alone [40–42]. Finally, it is possible that other bioactive lipids, such as PGE2-glycerol ester [43], are altered in the CNS of MAGL-disrupted animals and make additional contributions to fever regulation.

In summary, our data show that MAGL is a major regulator of fever in mice and put forth MAGL inhibitors as a potential class of antipyretic drugs.
